# Statistical Tests for Force Inference in Heterogeneous Environments

**DOI:** 10.1038/s41598-020-60220-1

**Published:** 2020-03-02

**Authors:** Alexander S. Serov, François Laurent, Charlotte Floderer, Karen Perronet, Cyril Favard, Delphine Muriaux, Nathalie Westbrook, Christian L. Vestergaard, Jean-Baptiste Masson

**Affiliations:** 1 Decision and Bayesian Computation, USR 3756 (C3BI/DBC) & Neuroscience department CNRS UMR 3751, Institut Pasteur, CNRS, Paris, France; 2 Infectious Disease Research Institute of Montpellier, CNRS UMR 9004, University of Montpellier, Montpellier, France; 3 Laboratoire Charles Fabry, Université Paris-Saclay, Institut d’Optique Graduate School, CNRS UMR8501, 91127 Palaiseau Cedex, France

**Keywords:** Single-molecule biophysics, Statistical methods, Statistics

## Abstract

We devise a method to detect and estimate forces in a heterogeneous environment based on experimentally recorded stochastic trajectories. In particular, we focus on systems modeled by the heterogeneous overdamped Langevin equation. Here, the observed drift includes a "spurious” force term when the diffusivity varies in space. We show how Bayesian inference can be leveraged to reliably infer forces by taking into account such spurious forces of unknown amplitude as well as experimental sources of error. The method is based on marginalizing the force posterior over all possible spurious force contributions. The approach is combined with a Bayes factor statistical test for the presence of forces. The performance of our method is investigated analytically, numerically and tested on experimental data sets. The main results are obtained in a closed form allowing for direct exploration of their properties and fast computation. The method is incorporated into TRamWAy, an open-source software platform for automated analysis of biomolecule trajectories.

## Introduction

Random walks are encountered throughout biology and other domains of science, and so is the associated inverse problem of inferring their properties from experimental data. Random walkers can be considered probes of their environment, and their recorded trajectories thus contain information on the properties of both the walker and its environment. In the context of biophysics, the random walkers are typically colloidal particles or biomolecules, but, in a general context, they may, for example, represent the motion along an abstract coordinate of a chemical reaction or the fluctuating price of a stock asset. Transport of biomolecules within cells^1^, conformational dynamics of proteins and RNA molecules^2^, diffusion of proteins on the DNA^3^, dynamics of nanosized objects in the cytosol^4^, dynamics of receptors in neurons^5–7^, complex random walks in mixed biological environments^8–11^, bacteria performing chemotaxis^12,13^, immune-cell dynamics^14–16^, and directionally persistent cell movement^17^ are all examples of cases where biologically relevant information can be extracted from recorded stochastic trajectories.

Empirical systems featuring biomolecule random walks are typically characterized by high heterogeneity, so the inverse problem often translates into inferring the properties of the heterogeneous environment from the trajectories of tracer molecules^18–31^. A paradigmatic model for random walks in such systems is the heterogeneous overdamped Langevin equation (OLE):

1 $$d{{\bf{X}}}_{t}=\frac{{\bf{f}}\left({{\bf{X}}}_{t}\right)}{\gamma \left({{\bf{X}}}_{t}\right)}dt+\sqrt{2D\left({{\bf{X}}}_{t}\right)}\circ d{{\bf{W}}}_{t},$$

which describes the continuous-time dynamics underlying a discrete-time recorded random walk^22^. Here **X**_*t*_ is the tracer’s position at time *t*, **f**(**X**) is the force acting on it in the point **X**, *D*(**X**) is its diffusivity, *γ*(**X**) is the viscous friction coefficient, and **W**_*t*_ is a Gaussian zero-mean continuous-time white noise process with uncorrelated increments and unit variance^32^. Owing to its simplicity, the OLE (1) is a popular model for biological random walks, providing an effective mesoscopic description of the dynamics^22^. As is often the case for models of biological systems, the OLE is empirically postulated rather than derived from the first principles, since this derivation is complex and requires taking into account many factors, such as the heterogeneity of the environment composition, presence of boundaries^33^ and hydrodynamic properties^34^, as well as possible noise correlations^35,36^. We refer the interested reader to (i) references^37,38^ for an in-depth discussion of the derivation of the OLE and for a microscopic model of crowded environments, to (ii) references^39–41^ for some approaches to the derivation of the equation of motion in media featuring diffusivity or temperature gradients, and to (iii) references^35,36,42^ for a discussion and experimental measurements of the extent to which Brownian noise is truly uncorrelated.

When the diffusivity *D*(x) varies in space, Eq. (1) is only well-defined after a convention for calculating the (stochastic) integral of the noise term has been defined^43^. Two well-known examples are the Itô and Stratonovich conventions. Each convention leads to a different extra "spurious” drift term, which is proportional to the diffusivity gradient^41,43,44^. This feature of the OLE is known as the Itô-Stratonovich dilemma^41,45–49^. For an experimental illustration of the presence of two physically different components see^50,51^.

Any stochastic convention can be used in the OLE to statistically describe a given experimental random walk. However, they are not equivalent to an external observer attempting to *interpret* the parameters of the random walk. Indeed, the two components of the drift in the OLE — the spurious force and the non-diffusive force — have a different physical nature. The spurious force is proportional to the diffusivity gradient, hence its value changes when the diffusivity, viscosity, or the temperature of the system change in space (time) or across systems. On the contrary, the non-diffusive component of the drift does not depend on the diffusivity and represents specific or non-specific interactions. The spurious force does not contribute to the equilibrium (Boltzmann) distribution.

Spurious forces are due to the interactions of the tracked particle with the surrounding thermal bath, while the non-diffusive forces represent its interactions with other objects or fields^45^. The separation of interactions between these two groups naturally depends (i) on the scale on which the system is analyzed and (ii) on which parts of the environment are included into the thermal bath. In Sect. 3.7 below, we show how in the same simulated system, the drift due to non-diffusive forces on the microscopic scale is perceived as a spurious force on the mesoscopic scale, when the contribution of individual interacting partners can no longer be identified.

For practical applications, it is thus important to develop a method allowing to distinguish between diffusive and non-diffusive forces or at least to develop a test allowing to confirm the presence of the non-diffusive forces on a given scale. The need for such approaches is further emphasized by the inaccessibility of the equilibrium distributions and of the exact boundary conditions at the nanometer scale in numerous biological setups.

Since the seminal work of Bachelier^52^, the inverse problem of drift and diffusivity inference from random walks has been attracting attention^53,54^, especially in financial applications^55,56^. In this article, we address a more specific problem of distinguishing between diffusive and non-diffusive forces, since the value of the latter is generally unknown. Although the spurious force is proportional to the diffusivity gradient *λ* ∇ *D*(**X**), it includes an unknown proportionality factor *λ*. It is known that for physical systems in equilibrium, described by Boltzmann distribution, *λ* = 1, but its value is not known in general for out-of-equilibrium systems^41,42,44,47–50,57,58^. Each value of *λ* represents specific symmetries of transition probabilities in these systems^46,58,59^.

Our goal is hence two-fold: (i) to develop a statistical test for the presence of non-diffusive forces, and (ii) to infer the posterior distribution for the intensity of the non-diffusive forces while taking into account all possible contributions of the "spurious” forces as well as experimental localization errors and motion blur. The method we introduce here is statistically robust to changes in the spurious force contribution in the OLE due, for example, to changes of the diffusivity or viscosity. We validate our approach on numerical trajectories and demonstrate its efficiency on experimental data.

## The Itô-Stratonovich Dilemma for the Inverse Problem

In this section we give a brief review of the Itô-Stratonovich dilemma^45^. Numerous discussions of the dilemma have been focused on choosing the appropriate integral convention for the forward problem of integrating the OLE in a particular system. In contrast, we here focus on how the dilemma affects the *inverse problem* of inferring the underlying physical parameters of a model from recorded data. To underline the generality of the problem, we rewrite the OLE (1) in the form of a general stochastic differential equation (SDE):

2 $$d{{\bf{X}}}_{t}={\bf{a}}({{\bf{X}}}_{t})dt+b({{\bf{X}}}_{t})\circ d{{\bf{W}}}_{t},$$

where a and *b* are differentiable functions of **X**_*t*_. We will refer to a and *b* as the drift and diffusivity respectively.

The integral of Eq. (2) is defined as the limit of Riemann sums

3 $${{\bf{X}}}_{T}={{\bf{X}}}_{0}+\mathop{lim}\limits_{N\to \infty }\left[\mathop{\sum }\limits_{i=0}^{N-1}a({{\bf{X}}}_{{\xi }_{i}})({t}_{i+1}-{t}_{i})+\mathop{\sum }\limits_{i=0}^{N-1}b({{\bf{X}}}_{{\xi }_{i}})({{\bf{W}}}_{{t}_{i+1}}-{{\bf{W}}}_{{t}_{i}})\right],$$

where each point *ξ*_*i*_ is chosen in the interval [*t*_*i*_; *t*_*i*+1_]. The standard conventions — Itô, Stratonovich-Fisk and Hänggi-Klimontovich — correspond to *ξ*_*i*_ = *t*_*i*_, *ξ*_*i*_ = (*t*_*i*_ + *t*_*i*+1_)/2 and *ξ*_*i*_ = *t*_*i*+1_ respectively^41,46^. More generally, *ξ*_*i*_ can be set to any point *ξ*_*i*_ = *t*_*i*_ + *λ*(*t*_*i*+1_ − *t*_*i*_) within the [*t*_*i*_; *t*_*i*+1_] interval. This allows one to rewrite Eq. (2) with any convention *λ* in the Itô form^60,61^:

4 $$d{{\bf{X}}}_{t}=\alpha ({{\bf{X}}}_{t},\lambda )dt+b({{\bf{X}}}_{t})d{{\bf{W}}}_{t},$$

where the total drift *α* is the sum of **a** and the spurious drift *λb*(**X**) ∇*b*(**X**):

5 $$\alpha ({\bf{X}},\lambda )\equiv {\bf{a}}({\bf{X}})+\lambda b({\bf{X}})\nabla b({\bf{X}}).$$

From the perspective of the forward problem, Eq. (5) shows that the often arbitrary choice of the value of *λ* influences the value of the drift *α* when a and *b* are fixed, — this is the essence of the Itô-Stratonovich dilemma^45^. In the context of the inverse problem, one is given fixed values of *α* and *b* estimated from the recorded trajectories, so different choices of *λ* result in different estimates of the non-diffusive drift a. If the chosen *λ* does not agree with its true value in the empirical system, the resulting estimate of a becomes biased.

We emphasize that we do not address here the forward problem, i.e. the question of finding the correct *λ* for a given system^41,45–49^. The correct *λ* values are often inaccessible in real biological systems. Instead, we aim to solve the inverse problem of whether non-diffusive forces are observed in the system and to infer their values if the appropriate value of *λ* cannot be determined. It is an inverse problem with an uncertainty in the underlying physical model. This ambiguity in *λ* may stem, for example, from the lack of *a priori* knowledge about the out-of-equilibrium fluxes in the system, noise correlations or the particle density distribution. In all cases, the method developed below allows one to obtain estimates of the non-diffusive forces and to circumvent the Itô-Stratonovich dilemma by marginalizing over all possible *λ* values. The estimates are robust to changes in the spurious force contribution in the OLE.

Above, we have formulated the main question of this paper from a physical point of view as that of inferring non-diffusive forces, when the correct *λ* is unknown. It is interesting to note that the same question can also be asked from a purely statistical point of view: *Given the OLE*, *does there exist a value of* 0 ≤ *λ* ≤ 1 *that would allow to describe the given system with zero non-diffusive forces* (a = 0)*?* This would allow to describe the same system with fewer parameters (*D* and *λ* instead of *D*, *λ*, a), thus minimizing the description length among all the descriptions proposed by the OLE family^62,63^.

From this point of view, the Bayes factor developed below is a Bayesian analog of the difference in the description lengths between the models with a ≠ 0 and a = 0 for the given data. It evaluates how much more efficient the non-diffusive-force description is, as compared to the spurious-force-only description of the same data. If the spurious-force description is preferred, as a byproduct, one can calculate the value of *λ* that provides the most efficient description of the data.

## The Bayesian Approach

Our goal is to discriminate between the following two nested hypotheses:

- *H*_0_: The only forces present are spurious forces due to heterogeneous diffusivity (the null hypothesis).
- *H*_1_: There are other, non-diffusive, forces acting on the random walker in addition to the spurious forces.

We use the Bayes factor to decide between these hypotheses^64^.

### The Bayes factor

According to Bayes’ rule^65^, the posterior probability Pr(*H*_*i*_ | *T*) of a hypothesis *H*_*i*_ given data *T* is

$${\rm{\Pr }}({H}_{i}| T)=\frac{p(T| {H}_{i})\pi ({H}_{i})}{p(T)}.$$

Here, *T* is a trajectory, $T\equiv {\{{{\rm{r}}}_{i}\}}_{i=1}^{n}$, or a set of trajectories; *p*(*T* | *H*_*i*_) is the marginal likelihood for the data *T* to be observed under the hypothesis *H*_*i*_; *π*(*H*_*i*_) is the prior probability of *H*_*i*_; and *p*(*T*) is the probability to observe *T* under either hypothesis. For the two competing hypotheses *H*_1_ and *H*_0_, the ratio of their posterior probabilities reads

$$\frac{{\rm{\Pr }}({H}_{1}| T)}{{\rm{\Pr }}({H}_{0}| T)}=\frac{p(T| {H}_{1})}{p(T| {H}_{0})}\frac{\pi ({H}_{1})}{\pi ({H}_{0})}.$$

The first fraction on the right-hand side is called the *Bayes factor* for *H*_1_ over *H*_0_^64^:

$$K\equiv \frac{p(T| {H}_{1})}{p(T| {H}_{0})}.$$

Each marginal likelihood *p*(*T* | *H*_*i*_) is calculated by marginalizing the corresponding conditional likelihood *p*(*T* | *θ*_*i*_, *H*_*i*_) over all model parameters:

$$p(T| {H}_{i})=\int d{\theta }_{i}\ p(T| {\theta }_{i},{H}_{i})\pi ({\theta }_{i}| {H}_{i}).$$

For *H*_0_, the likelihood *p*(*T* | *θ*_0_, *H*_0_) thus depends on 3 parameters: *θ*_0_ = {*b*^2^, g, *λ*}, where *b*^2^ is the diffusivity and g ≡ ∇ *b* is the diffusivity gradient. For *H*_1_, the likelihood *p*(*T* | *θ*_1_, *H*_1_) additionally includes the drift a, so *θ*_1_ = {*b*^2^, g, *λ*, a}. Note that we treat g as independent from *b*^2^, which allows us to obtain the results in the analytical form. This assumption is further discussed in Appendix A1.

### Likelihood

The likelihood *p*(*T* | *θ*_*i*_, *H*_*i*_) is obtained as the fundamental solution of the Fokker-Planck equation corresponding to the OLE (2). However, it cannot in general be obtained analytically. Instead, one can approximate it locally by assuming that *α* and *b* are constant within small spatial domains^20,21^. In this case, the likelihood of observing a set of displacements {*Δ***r**} inside a given domain is^21^:

6 $$p(\{\Delta {\rm{r}}\}| \alpha ,{b}^{2})={(2\pi {b}^{2}\Delta t)}^{-nd/2}\exp \left(-\frac{n{\left(\overline{\Delta {\bf{r}}}-\alpha \Delta t\right)}^{2}+nV}{2{b}^{2}\Delta t}\right).$$

Here the mean displacement $\overline{\Delta {\rm{r}}}\equiv {\sum }_{i=1}^{n}\Delta {{\rm{r}}}_{i}/n$ and the biased sample variance $V\equiv {\sum }_{i=1}^{n}| \Delta {{\rm{r}}}_{i}-\overline{\Delta {\rm{r}}}{| }^{2}$/*n* are the sufficient statistics of the model^65^, and *d* is the number of dimensions. The equations below are valid for *d* = 1 and *d* = 2, but the framework can also be extended to *d* = 3.

Note that calculations would be similar if one relaxed the approximation of the locally constant values of *α* and *b*. Computations would be performed numerically but the analytical explorations such as those of Appendix A3 would not be possible. Meanwhile, the assumption of bin independence is paramount to the presented method.

### Priors

The likelihood (6) belongs to the exponential family^65^. Therefore, a natural choice for the prior is a conjugate prior for the parameters a and *b*^2^. Among other advantages, conjugate priors provide a closed form of the posterior distribution. We furthermore assume a factorized form for the prior distributions for *λ* and the diffusivity gradient g:

$$\pi (a,\lambda ,{\rm{g}},{b}^{2}| {H}_{1})\approx \pi (a,{b}^{2}| \lambda ,{H}_{1})\ \pi (\lambda )\ \pi ({\rm{g}}).$$

We have no *a priori* information available about the true value of *λ* other than that 0 ≤ *λ* ≤ 1, so we use the flat prior *π*(*λ*) ≡ 1. The diffusivity gradient prior is approximated by a delta function $\pi ({\rm{g}})\equiv \delta ({\rm{g}}-\widehat{{\rm{g}}})$ centered around its maximum *a posteriori* (MAP) value $\widehat{{\rm{g}}}$. Details of $\widehat{{\rm{g}}}$ estimation are given in Appendix A1.

Under *H*_1_, the full conjugate prior is then (cf. (6)):

7 $$\pi ({\bf{a}},\lambda ,{\rm{g}},{b}^{2}| {H}_{1})\equiv {A}_{d}\ {\left({b}^{2}\Delta t\right)}^{-d{n}_{\pi }/2}\exp \left(-\frac{{n}_{\pi }{((a+\lambda {\rm{g}})\Delta t-{\mu }_{\pi })}^{2}+{n}_{\pi }{V}_{\pi }}{2{b}^{2}\Delta t}\right)\delta ({\rm{g}}-\widehat{{\rm{g}}}),$$

where ${A}_{d}\equiv {({n}_{\pi }/(2\pi ))}^{d/2}{({n}_{\pi }{V}_{\pi }/2)}^{m(d)}\Delta {t}^{d+1}/\Gamma (m(d))$; *m*(*d*) ≡ *d*(*n*_*π*_ − 1)/2 − 1; *μ*_*π*_, *V*_*π*_ and *n* are the parameters of the prior (called hyper-parameters).

The models *H*_0_ and *H*_1_ are nested models. In such case, it is common practice to obtain the *H*_0_ prior by integrating the *H*_1_ prior over a^64^:

8 $$\pi (\lambda ,{\rm{g}},{b}^{2}| {H}_{0})={A}_{d}{\left(\frac{2\pi }{{n}_{\pi }\Delta {t}^{2}}\right)}^{d/2}{\left({b}^{2}\Delta t\right)}^{\frac{d(1-{n}_{\pi })}{2}}\exp \left(-\frac{{n}_{\pi }{V}_{\pi }}{2{b}^{2}\Delta t}\right)\delta ({\rm{g}}-\widehat{{\rm{g}}}).$$

We further set the hyper-parameters to maximally favor the null model. More specifically, *n*_*π*_ acts as an effective number of prior observations. The least constraining prior is obtained by setting *n*_*π*_ = 4 for 1D data and *n*_*π*_ = 3 for 2D, which are the minimal number of observations, for which the prior is proper (normalized). Furthermore, we center the prior on zero force by setting *μ*_*π*_ = *λ*g*Δ*_*t*_. The remaining hyper-parameter *V*_*π*_ defines the prior distribution for the diffusivity. Sensitivity of the results to *u* ≡ *V*_*π*_/*V* is explored in Appendix A2.

### Model evidence and the Bayes factor

The evidence for the *H*_1_ and *H*_0_ models is the central ingredient in the Bayes factor. Given the likelihood *p*({*Δ***r**} | *α*(a, *λ*), *b*^2^) (Eq. (6)) and prior *π*(a, *λ*, g, *b*^2^ | *H*_1_) (Eq. (7)), the evidence for *H*_1_ is calculated by marginalizing *p*({*Δ***r**} | *α*(a, *λ*), *b*^2^)*π*(a, *λ*, g, *b*^2^ | *H*_1_) over all the parameters *θ*_1_ = {*a*, *b*^2^, *g*, *λ*}. This gives

9 $$p(\{\Delta {\rm{r}}\}| {H}_{1})=\frac{{A}_{d}{C}_{d}}{{(n+{n}_{\pi })}^{d/2}}{\int }_{0}^{1}d\lambda {(nV+{n}_{\pi }{V}_{\pi }+\frac{n{n}_{\pi }}{n+{n}_{\pi }}{(\overline{\Delta {\bf{r}}}-\lambda {\rm{g}}\Delta t)}^{2})}^{-\kappa (d)},$$

where $${C}_{d}={2}^{\kappa (d)}\Gamma (\kappa (d)){(2\pi )}^{\frac{d(1-n)}{2}}\Delta {t}^{-d-1}$$ and *κ*(*d*) ≡ *d*(*n* + *n*_*π*_ − 1)/2 − 1.

For *H*_0_, the likelihood *p*({*Δ***r**} | *α*(*λ*), *b*^2^) is given by Eq. (6) with *α*(*λ*) = *λb* ∇*b*, and the prior *π*(*λ*, g, *b*^2^ | *H*_0_) by Eq. (8). Marginalization of *p*({*Δ***r**} | *α*(*λ*), *b*^2^)*π*(*λ*, g, *b*^2^ | *H*_0_) over *θ*_0_ = {*b*^2^, g, *λ*} gives

10 $$p(\{\Delta {\rm{r}}\}| {H}_{0})=\frac{{A}_{d}{C}_{d}}{{n}^{d/2}}{\int }_{0}^{1}d\lambda {(nV+{n}_{\pi }{V}_{\pi }+n{(\overline{\Delta {\bf{r}}}-\lambda {\rm{g}}\Delta t)}^{2})}^{-\kappa (d)}.$$

Expressions (9) and (10) let us finally calculate the marginalized Bayes factor *K*^M^, which takes into account all possible values for the unknown parameter *λ*. For comparison, we also provide the Bayes factor *K*(*λ*) for fixed-*λ* inference procedures (Itô, Stratonovich or Hänggi), which is calculated in the same manner:

11 $${K}^{{\rm{M}}}={\eta }^{d}\frac{{\int }_{0}^{1}d\lambda {\left[v+{\eta }^{2}{({\zeta }_{{\rm{t}}}-\lambda {\zeta }_{{\rm{sp}}})}^{2}\right]}^{-K(d)}}{{\int }_{0}^{1}d\lambda {\left[v+{({\zeta }_{{\rm{t}}}-\lambda {\zeta }_{{\rm{sp}}})}^{2}\right]}^{-K(d)}},\quad K(\lambda )={\eta }^{d}{\left[\frac{v+{\eta }^{2}{({\zeta }_{{\rm{t}}}-\lambda {\zeta }_{{\rm{sp}}})}^{2}}{v+{({\zeta }_{{\rm{t}}}-\lambda {\zeta }_{{\rm{sp}}})}^{2}}\right]}^{-K(d)}.$$

All the integrals appearing in Eqs. (9–11) are 1D integrals that are numerically evaluated using the trapezoid rule.

The natural parameter combinations appearing in Eq. (11) are: (i) ${\zeta }_{{\rm{t}}}\equiv \overline{\Delta {\rm{r}}}/\sqrt{V}$, the signal-to-noise ratio for the total force in a single displacement; (ii) ${\zeta }_{{\rm{sp}}}\equiv \widehat{{\rm{g}}}{\Delta }_{t}/\sqrt{V}$, the signal-to-noise ratio for the spurious force in a single displacement; (iii) $\eta \equiv \sqrt{{n}_{\pi }/(n+{n}_{\pi })}$, the relative strength of the prior compared to the observed data; (iv) *v* ≡ 1 + *n*_*π*_*V*_*π*_/(*nV*), a weighted ratio of jump variances in the prior and in the data.

**Figure 1:**
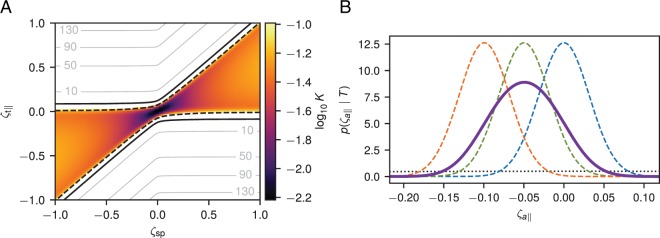
(**A**) Marginalized Bayes factor *K*^M^ for the presence of forces in a 2D system as a function of the signal-to-noise ratios *ζ*_*sp*_ and *ζ*_∥_ (see text). Black lines show the Bayes factor levels ${{\rm{\log }}}_{10}{K}^{{\rm{M}}}=1$ (solid) and ${{\rm{\log }}}_{10}{K}^{{\rm{M}}}=-1$ (dashed). Gray lines mark levels of Bayes factor for ${{\rm{\log }}}_{10}{K}^{{\rm{M}}}\ge 10$. The color map shows Bayes factor values for ${{\rm{\log }}}_{10}{K}^{{\rm{M}}}\le -\,1$. *K*^M^ behavior is qualitatively similar for other parameter values. (**B**) Force posteriors for a 2D system with *ζ*_*sp*_ = 0.1 and *ζ*_t_ = 0. Posterior distributions obtained for Itô (dashed blue line), Stratonovich (dashed green), Hänggi (dashed orange) and marginalized (solid magenta) approaches are shown alongside their common prior (dotted black). In both panels, the number of recorded displacements is *n* = 500, the prior hyperparameter *u* = 1.0, the perpendicular component of the total force *ζ*_*t*⊥_ = 0, and the localization error ${\sigma }_{L}^{2}=0$.

Figure 1A plots the marginalized Bayes factor *K*^M^ (11) as a function of *ζ*_*sp*_, and of the component of the total force *ζ*_∥_ parallel to *ζ*_sp_. The lowest values of *K*^M^ are achieved in the region 0 ≤ *ζ*_∥_/*ζ*_*sp*_ ≤ 1. The value of *K*^M^ changes relatively little within this region but grows rapidly at its boundary. The absolute minimum of *K*^M^ is achieved for *ζ*_*sp*_ = 0 and *ζ*_∥_ = 0 with $\min \ {K}^{{\rm{M}}}=\min \ K(\lambda )={\eta }^{d}{[(v+{\eta }^{2}{{\zeta }_{t\perp }}^{2})/(v+{{\zeta }_{t\perp }}^{2})]}^{-\kappa }$. A mathematical analysis of Eq. (11) is provided in Appendix A3, where it is shown that non-diffusive forces cannot in principle be detected in certain intervals of *ζ*_sp_, *ζ*_t_ regardless of the number of collected data points. Appendix A4 extends the Bayes factors (11) to the experimentally relevant case with localization errors and motion blur.

### Force posterior

When *H*_1_ is met, we can infer the value of the non-diffusive force by marginalizing the force posterior over all possible values of *λ*:

12 $$p({\zeta }_{a}| T)\propto {\int }_{0}^{1}d\lambda {\left[v+\frac{{\eta }^{2}}{1-{\eta }^{2}}{\zeta }_{a}^{2}+{({\zeta }_{{\rm{t}}}-{\zeta }_{a}-\lambda {\zeta }_{{\rm{sp}}})}^{2}\right]}^{-K(d)-d/2},$$

where a signal-to-noise ratio for the force *ζ*_*a*_ ≡ aΔ*t*/$\sqrt{V}$ was introduced. Figure 1B plots an example force posteriors obtained with the marginalized method and with fixed-*λ* inference schemes. The wider marginalized method posterior takes into account all possible *λ* values. Appendix A5 demonstrates that in contrast to the fixed-*λ* posteriors, the marginalized posterior is in general non-symmetric.

### Numerical results

**Figure 2:**
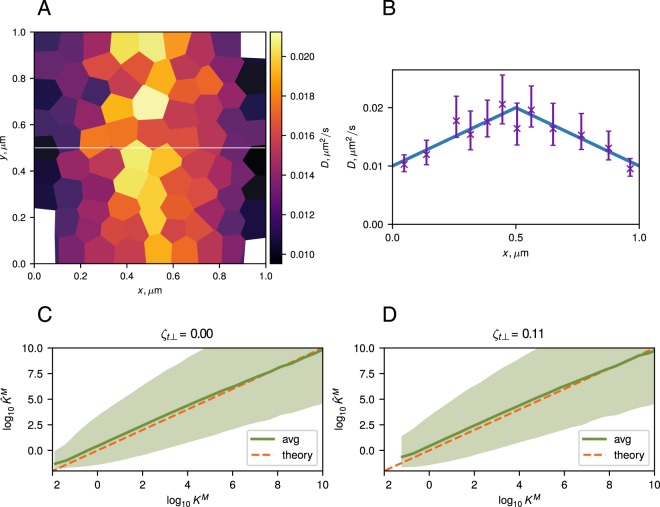
(**A**) A diffusivity map inferred using the TRamWAy software platform^66^. The white line *y* = 0.5 *μ*m indicates the axis along which the diffusivity profile is plotted in (**B**). (**B**) 1D diffusivity profile showing the true diffusivity (solid blue line) and *D* values inferred from a single simulation (magenta crosses). Error bars show 95% confidence intervals (CI) calculated from the diffusivity posterior (see Appendix A1). (**C**,**D**) Inferred values of the marginalized Bayes factor ${{\rm{\log }}}_{10}{\widehat{K}}^{{\rm{M}}}$ as a function of its expected value: sliding average (solid green line) over a window of constant width 0.5 on the logarithmic scale and the corresponding 95% CI (shaded green region). The observed dependency is centered around the identity line (${\widehat{K}}^{{\rm{M}}}={K}^{{\rm{M}}}$, dashed orange) indicating that the inferred Bayes factors are approximately unbiased for both the case when the total force is parallel to the spurious force (*ζ*_*t*⊥_ = 0, (**C**)) and when it is not (*ζ*_*t*⊥_ = 0.11, (**D**)). The Bayes factors were inferred in individual domains shown in (**A**), with an average of ~123 individual domains per trial. In each trial, the spatial tessellation was performed independently based on the relative particle density. The calculations were repeated across 100 trials for each value of *ζ*_∥_ out of the analyzed range (see Appendix A6).

The performance of the marginalized method was investigated on simulated trajectories. Random trajectories were simulated in a 2D box with periodic boundary conditions, a uniform total force, and a triangular diffusivity profile along the *x* axis (Fig. 2A,B). Other simulation parameters are given in Appendix A6. For each trial, the simulated trajectories were then analyzed using the TRamWAy software platform^66^ and following a procedure similar to the one used in reference^20,28^ and consisting of (i) individual spatial tessellation in each trial; (ii) assignment of recorded displacements to spatial domains; (iii) inference of *ζ*_sp_ and *ζ*_t_ in each domain; (iv) calculation of the Bayes factor in each domain.

The marginalized Bayes factor ${\widehat{K}}^{{\rm{M}}}$, inferred in each domain, was then plotted against its expected value *K*^M^ to test the accuracy of the method (Fig. 2C,D). The figure shows good correspondence between the inferred Bayes factor and the expected Bayes factor. 95% confidence intervals (CIs) show the extent of the deviation of the results from the true values due to the stochastic nature of the simulated trajectories.

### Microscopic model of heterogeneous diffusivity

**Figure 3:**
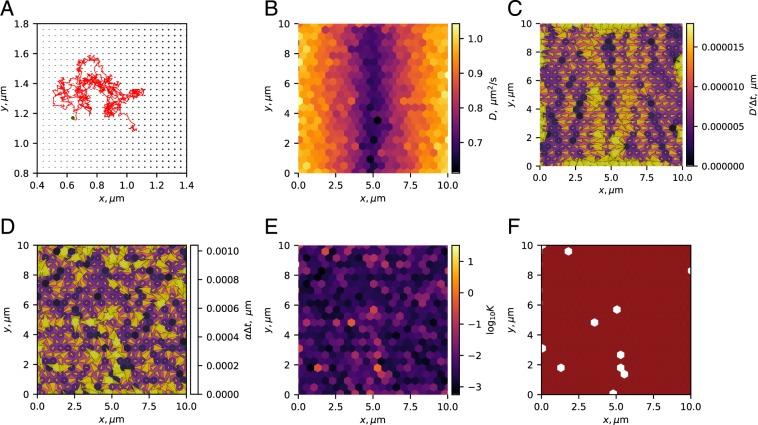
Simulations of mesoscopic changes in particle diffusivity due to microscopic crowding. Inference results for particles diffusing in the presence of a lattice of immobile beads of various radius. (**A**) A zoom-in on a small 1 × 1 *μ*m^2^ section of the simulated 10 × 10 *μ*m^2^ system. The radius of the immobilized beads located in the nodes of a lattice changes with *x* leading to an effective diffusivity gradient on a larger scale (cf. (**B**,**C**). A sample trajectory of 1000 jumps of a single diffusing particle, with a green circle indicating the origin point. In total, 1000 independent diffusing particles were simulated. (**B**) Inferred diffusivity. (**C**) Inferred diffusivity gradient. Arrows indicate the direction and the strength of the gradient, also represented by the bin color. (**D**) Inferred drift. Arrows indicate the direction and the strength of the drift, also represented by the bin color. (**E**) Estimated Bayes factor. (**F**) Thresholded Bayes factor. Color code: green (non-spurious force, ${{\rm{\log }}}_{10}K\ge 1$), red (spurious force only, ${{\rm{\log }}}_{10}K\le -1$), white (insufficient evidence, $| {{\rm{\log }}}_{10}K|  < 1$). Values of *D*, ${D}^{{\prime} }$ and *α* in the plots (**B**–**D**) were clipped at high values around the 9th decile to allow for a clearer visualization. Simulation details are provided in Appendix A7.

The next simulation was performed with two goals: (i) to illustrate how spurious forces may originate from crowding at the molecular scale, and (ii) to illustrate a case, wherein our developed statistical test successfully indicates the absence of non-diffusive forces. For this purpose, we simulated free diffusion of particles with no microscopic drift within a square region with periodic boundaries and with impenetrable immobile beads evenly spaced on a square lattice (Fig. 3A), similar to schemes suggested in^67–69^. The microscopic diffusivity of the particle was the same throughout the system. A spatial variation in the radii of the immobile beads created a spatial variation in the effective diffusivity on a much larger “mesoscopic” scale, where each analysis bin included ~100 small beads (Fig. 3B). As a result, recordings at the mesoscopic scale exhibit a diffusivity gradient (Fig. 3C), which contributes to the drift observed on the same scale (Fig. 3D). Note that at long time scales, the system is in physical equilibrium, although particles experience a stationary non-zero drift. Simulation details and parameters are provided in Appendix A7.

The diffusivity gradient contribution to the drift is the spurious force, its exact value depends on *λ*. Assuming the value of *λ* is unknown, one can use the Bayes factor test developed above to estimate the *a posteriori* likelihood of that the observed drift is due to non-spurious forces (Fig. 3E). In our simulation, the inferred Bayes factors were small (${{\rm{\log }}}_{10}K < -1$) in most parts of the region, supporting the claim that only spurious forces were present (Fig. 3F). Statistical noise in several bins resulted in weaker evidence, which did not let us draw statistically significant conclusions in those zones. These results confirm the capacity of the method to detect spurious forces. Its capacity to detect non-spurious forces will be illustrated in the next section.

This simulation captures one possible microscopic mechanism behind the observation of a diffusivity gradient on the mesoscopic scale in biological systems. However, note that the homogeneous composition required for a uniform microscopic diffusivity is probably achievable in the biological systems only on the molecular scale (10^−9^ m and smaller). On this scale though, it is not clear whether the diffusivity itself is well-defined, since by definition it is the result of the action of millions of individual molecules and Fick’s law describes an intrinsically mesoscopic phenomenon.

Other microscopic mechanisms for the diffusivity gradient include (i) confinement, wherein it was shown that the diffusivity in a homogeneous system changes with the distance to a wall^33,41,50,70^, (ii) corralled motion^71^, (iii) hydrodynamic coupling to other objects in the medium^34,72^, (iv) temperature gradients^44,73,74^, or (v) intermittent trapping^75^.

## Applications

The developed method was tested on two experimental systems. The first one was a well-controlled setup of a bead in the optical tweezers. The second one was a complex biological process of HIV virion assembly in a T cell^76^, where the OLE is potentially only an approximation to the true biomolecule dynamics (ignoring inertial effects, colored noise or memory of the previous states).

### Optical tweezers

**Figure 4:**
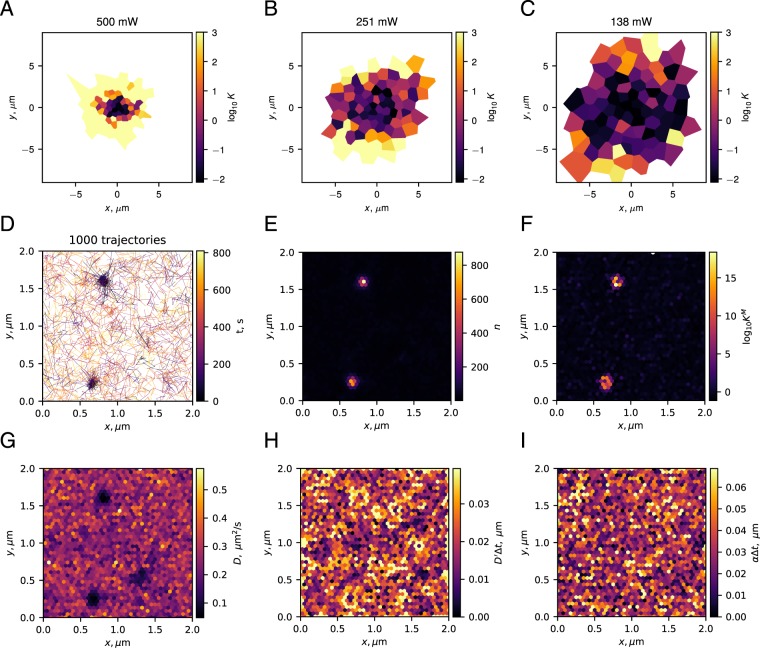
(**A**–**C**) Bayes factors for the presence of non-spurious forces inferred from experimentally-recorded trajectories of a bead trapped in the optical tweezers at 3 different levels of laser power: (**A**) 500 mW, (**B**) 251 mW, (**C**) 138 mW. For a better visual representation, all values of ${{\rm{\log }}}_{10}{K}^{{\rm{M}}} > 3$ (very strong evidence for *H*_1_) are shown as ${{\rm{\log }}}_{10}{K}^{{\rm{M}}}=3$. Each domain contained strictly between 390 and 410 recorded displacements. (**D**–**I**) Bayes factors analysis for single-molecule-dynamics of the *Gag* protein during the assembly of HIV VLPs in human T cells. (**D**–**I**) All panels show the same 2 *μ*m × 2 *μ*m patch of a T cell membrane. (**D**) 1000 trajectories randomly chosen among the 12 825 trajectories of the data set. (**E**) Number of displacements *n* attributed to each domain. (**F**) Common logarithm of the marginalized Bayes factor *K*^M^. Note the high values of ${{\rm{\log }}}_{10}{K}^{{\rm{M}}}$ where *Gag* particles cluster. (**G**) Inferred diffusivity field. (**H**) Absolute value of the inferred diffusivity gradient. (**I**) Absolute value of the inferred total force. In panels (H,I), large values have been clipped around the 9th decile to increase plot readability.

Optical tweezers combine physical trapping of the bead with local laser heating of the medium, leading to a heterogeneous diffusivity field. Therefore, the heating effect and the ensuing spurious forces may interfere with the inferred trapping potential. Figure 4A–C compares the results of Bayes factor calculations for the same system subjected to three different laser powers. The tessellation procedure was designed to assign the same number of jumps to each domain. In all 3 cases, the particle is confined and the Bayes factor favors the presence of forces (${{\rm{\log }}}_{10}{K}^{{\rm{M}}} > 1$) in a large number of domains, which form a connected region. With the decrease of the laser power, the confinement at the center of the trap becomes more shallow, so that the statistical test only detects confining forces on the trap border.

### Assembly of HIV-1 Virus-Like particles

The HIV virus-like-particle (VLP) assembly experiments that provided the data are described in reference^77^. The VLPs derive from the human immunodeficiency virus type 1 (HIV-1), but are immature and deprived of envelope proteins. One of their main components is the group-specific antigen (*Gag*) protein. It is a viral structural protein produced by the virus that anchors and oligomerizes at the plasma membrane of the host T cells, eventually assembling into a VLP^76^. In the experiments, the HIV-1 *Gag* precursor was genetically modified to contain a photoactivable fluorescent tag mEOS2 protein. It allowed to record VLP assembly in human CD4 T cells using single-particle tracking photoactivated localization microscopy (sptPALM)^77,78^. Several VLPs can assemble in parallel in the same observation region.

The TRamWAy software platform was used to tessellate the observation region and infer maps of diffusivities (Fig. 4G) and drift^20,66^. The Bayes factor map was then computed. The localization uncertainty was *σ*_*L*_ = 30 nm, requiring corresponding corrections to the Bayes factor (Appendix A4). Inference results and Bayes factors for a 2 *μ*m × 2 *μ*m zone of one T-cell membrane are shown in Fig. 4D–F.

Plots of the trajectories, the density of the recorded points and the diffusivity (Fig. 4D,E,G) indicate that there are two regions of interest (ROI) in the data set. However, the plots of the diffusivity gradient and the drift (Fig. 4H,I) suggest that the two parameters are of the same scale, hence it is not *a priori* clear, whether the localization of the particles is due to non-diffusive or spurious forces. Only the calculation of the Bayes factors for these regions allowed us to confirm that it is not solely due to heterogeneities in the diffusivity but rather to non-spurious forces (${{\rm{\log }}}_{10}(K)\gg 1$, Fig. 4F).

Some other individual domains in Fig. 4F bear evidence of a force with rather high Bayes factors (${{\rm{\log }}}_{10}(K)\ge 1$). In such a complex system, the high *K* values in these individual domains may stem from local membrane activity, failed capsid assembly^77^ or be false detection. In the rest of the region, the Bayes factor is $| {{\rm{\log }}}_{10}(K)|  < 1$ meaning neither of the models is favored at the chosen level of statistical significance.

**Figure 5:**
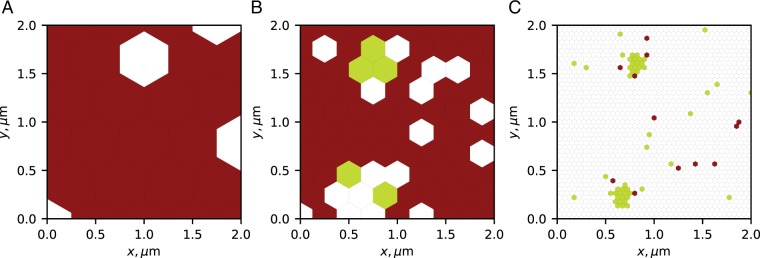
Thresholded Bayes factors for the VLP data set inferred at three different spatial scales. The average distance between bins was set to 0.5 *μ*m (**A**), 0.25 *μ*m (**B**), 0.05 *μ*m (**C**). Panel (C) demonstrates the same mesh as in Fig. 4D–I. Color code: green — non-spurious force, ${{\rm{\log }}}_{10}K\ge 1$; red — only spurious force, ${{\rm{\log }}}_{10}K\le -1$; white — insufficient evidence, $| {{\rm{\log }}}_{10}K|  < 1$.

As demonstrated in the simulation of Sect. 3.7, the results of any inference procedure depend on how the spatial scale on which the analysis is performed, corresponds to the internal scale of the observed system. An illustration of this fact for the VLP data can be seen in Fig. 5. Here, our statistical test was applied to the same VLP data set on three different spatial scales. When the bins are much larger than the typical structures present in the biological system (Fig. 5A, 0.5 *μ*m), the interactions are averaged out and our statistical test confirms the absence of interactions or is inconclusive. On the scale of the structures (Fig. 5B, 0.25 *μ*m), one may identify the potential regions of interest, but is unable to resolve their internal structure. When the bins are smaller than the regions of interest (Fig. 5C, 0.05 *μ*m), given enough data, the internal structure of the regions can be resolved. At even smaller scales (not shown), when few points are available per bin, one starts losing the connectivity of the regions of interest, and the statistical tests becomes inconclusive or (by design) favor the model with only spurious forces (*H*_0_). We suggest that one should aim for a scale smaller than the scale of the analyzed structure, but maintain enough points per bin to reach statistically significant conclusions.

## Discussion

In this paper, we introduced a method to address the inverse problem for the spatially heterogeneous OLE that is robust in regards to changes in the spurious-force contribution. We leveraged Bayesian inference and Bayesian model comparison to account for the uncertainty in the values of the spurious force caused by a heterogeneous diffusivity field. The method provides a test for the presence of non-diffusive forces and returns the values of the non-diffusive forces and diffusivity.

The marginalized posterior takes into account the error in the inferred forces due both to stochastic errors and to possible spurious forces when the true value of *λ* is unknown. The expression for the Bayes factor was derived in a closed form, allowing for identification of natural parameters associated with the dynamics, namely, the signal-to-noise ratios for the total force (drift) and spurious forces, *ζ*_t_ and *ζ*_sp_, and the relative strength of the localization uncertainty $4{\sigma }_{L}^{2}$/(*nV*). Interestingly, we showed that under some configurations, the discrimination between active and spurious forces is impossible without introducing additional assumptions.

As for any statistical method, a prerequisite for our method is that one observe the trajectories on the "right” spatial and temporal scales, which depend on the individual system. In particular, the spatial tessellation employed here should be constructed on the appropriate spatial scale, i.e. finer than the spatial heterogeneities of interest and coarse enough to provide sufficient measurements in each mesh domain (as discussed above). Another condition required by our method is that the number of points per bin be >4 in 1D and >3 in 2D, which are the equivalent numbers of points contained in the prior. Otherwise, due to the choice of *μ*_*π*_, the model with only spurious forces is likely to always be favored. Our experience with the method indicates that for the biological data we tested, *n* ≥ 20 typically provides a reasonable compromise between the spatial and statistical resolution.

The VLP example demonstrated successful utilization of the method for the detection of biological activity. The test was applied in an unsupervised way, which makes it useful for automatic analysis of single-molecule dynamics. In general, however, the results may depend on the spatial meshing. For the VLP data set, we had the advantage of *a priori* knowing the characteristic spatial scale of the underlying biological processes^77^. In a general case, one may need to sample multiple spatial scales in an attempt to optimize the detection. An optimal mesh scale in this case can be seen as a trade-off between increasing statistical significance (by getting more data per domain) and increasing resolution (by reducing the domain size).

Potential ways to circumvent this fundamental trade-off of spatial versus statistical resolution could be to regularize the inference of the diffusivity and drift fields^20,79^ or to cluster the regions with similar Bayes factor values based on a certain rule. However, the former approach induces correlations between the results inferred in different domains making analytical calculations intractable and hindering interpretation of the results. The main difficulty with the latter approach consists in defining the appropriate clustering criterion and in accounting for how the uncertainty in the individual Bayes factors propagates to the Bayes factors of the clusters.

One should also keep in mind that the validity of the main result (11) relies on the assumption that the diffusivity *b* is smooth enough, so that the gradient  ∇*b* exists on the spatial scale on which the system is experimentally probed. Additionally, we stress that *α* and *b* are mesoscopic quantities and their values may change depending on the analyzed scale^80^. In practice, the choice of the spatial and temporal resolutions for the analysis is limited by the particular experimental setup and the properties of the biological system.

The Bayesian approach that we proposed here is general and not limited to the OLE equation. The ambiguity of the stochastic integration is encountered in numerous other scientific fields involving stochastic equations with multiplicative heterogeneous noise. The effect is usually ignored and an arbitrary standard convention is used. The marginalized method allows us to avoid arbitrarily choosing an integral convention in the absence of system-specific information, therefore providing more robust results.

The marginalized method code is available as a module of the open source project TRamWAy^81^ and the microscopic crowding simulation code is available at^82^. Two Jupyter notebooks are provided as illustration of the module interface^66^.
